# Techniques of Skin Biopsy and Practical Considerations

**DOI:** 10.4103/0974-2077.44174

**Published:** 2008

**Authors:** Urmila Nischal, Nischal KC, Uday Khopkar

**Affiliations:** 1 *Consultant Dermatologist, Kaya Skin Centre, MG Road, Banglore, Karnataka, India*; 2 *Department of Dermatology, Adichunchanagiri Institute of Medical Sciences and Research, BG Nagar, Karnataka, India*; 3 *Department of Dermatology, Seth GS Medical College and KEM Hospital, Mumbai, India*

**Keywords:** Skin biopsy, mucosal biopsy, techniques, complications

## Abstract

Skin biopsy is a safe, easy and out-patient procedure of diagnostic and academic relevance. There are various methods of performing skin biopsy depending on the size of lesion, suspected clinical diagnosis and site of lesion. Although biopsy is usually a safe procedure, complications such as bleeding, infection and scarring may occasionally be encountered while performing biopsy in an out-patient with basic infrastructure. This article details the various techniques of skin biopsy, their indications and practical steps to curtail complications arising from the procedure.

## INTRODUCTION

Skin biopsy is an invaluable tool in the diagnostic armamentarium of a dermatologist. It not only helps in diagnosis in cases of dilemma but also provides an opportunity to find something unusual in routine practice. In the era of evidence-based medicine, consumer activism and litigations, skin biopsy helps in ensuring documentary evidence for the diagnosis made and the basis for the treatments started. Hence, it is important to have the basic knowledge of techniques of skin biopsy so as to derive the maximum from the procedure.

## CHOOSING THE LESION FOR BIOPSY

Choosing the right lesion for biopsy and adapting the right technique for performing biopsy ensures better interpretation of the biopsy. It is a classical dictum to choose the classical, well-formed, non-modified (by scratching or any topical application) lesion. However, there are exceptions to this dictum, e.g. biopsy of an early lesion is preferred in Henoch Schönlein purpura (to demonstrate leukocytoclasis and IgA deposition in vessel wall) and dermatitis herpetiformis (to demonstrate collection of neutrophils at the tips of dermal papilla). Also, when a subepidermal blister is suspected, it is better to avoid an old blister because reepethilialization may make a subepidermal blister look like an intraepidermal blister. Frictional sites like knees, elbows and the dorsal aspect of joints are avoided because secondary changes interfere with the interpretation of skin biopsy. When the patient has polymorphic lesions, never hesitate to take more than one biopsy.

## LOCAL ANAESTHESIA FOR SKIN BIOPSY

Lignocaine is the most commonly used local anaesthetic agent used for skin biopsy. Local anaesthesia is best achieved by infiltration, ring or field block or peripheral nerve block. Topical anaesthesia with eutectic mixture of local anesthetics (EMLA) has been used by some. Anaesthesia achieved with EMLA after 2 h of occlusion is about 5-mm deep.[1] This depth is quite sufficient for performing skin biopsy on sites like the flexural aspect of extremities, chest, abdomen, face and genitals. However, in areas where the epidermis is thick (palms, soles) or the dermis is thick (back) or a deep biopsy with good amount of subcutaneous tissue is required, as in cases of panniculitis, topical anaesthesia may be less effective.

Lignocaine with adrenaline prolongs the duration of anaesthesia in addition to curbing blood loss during the procedure. The use of adrenaline for digital block is a controversy. Evidence supports that, contrary to the long-held view, unless the person has peripheral vascular disease, connective tissue diseases, Raynaud’s disease or antiphospholipid syndrome, lignocaine with adrenaline may be used for digital anaesthesia.[2–5]

## TECHNIQUES OF SKIN BIOPSY

There are various techniques of performing a skin biopsy and any particular technique chosen is based on the type of lesion, site of lesion and also on the proficiency of the dermatologist.

### Punch biopsy

This is the most widely used technique for skin biopsy. This technique can be used both for diagnostic as well as therapeutic purposes (viz, punch excision of small pyogenic granuloma, verruca, tattoo). This method can be employed for any solid lesion and small vesicle that can be contained within the punch.

The lesion or area to be biopsied is punched out using a disposable or sterilizable punch of varying sizes. For non-facial lesions, a 4-mm punch is sufficient; however, in granulomatous conditions or conditions with atypical features, biopsies of 5 mm or more are preferable. It is advisable not to take less than 3 mm biopsies as vital features may be missed in such small biopsies.

**Figure 1:**
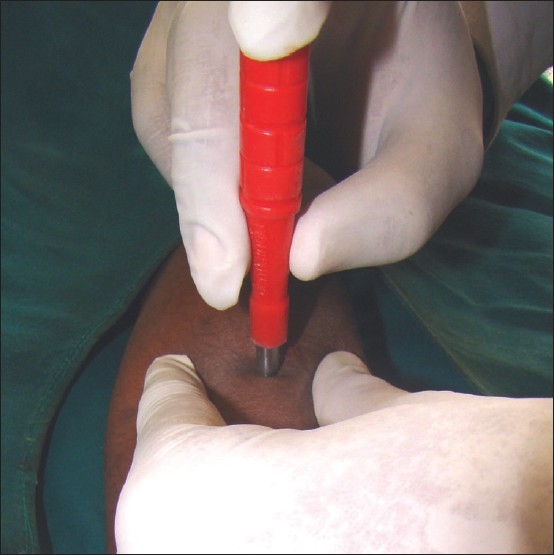
Proper technique of holding the punch for performing punch biopsy

The area is anaesthetized and the skin is stretched in a direction perpendicular to the resting skin lines. This maneuver converts the round defect in the skin to oval and hence reduces the chances of dogear formation during suturing. The punch is held with three fingers, with the thumb and the middle finger supporting and rotating the punch while the index finger stabilizes and exerts pressure on the punch [Figure 1]. The punch is pushed into the skin by rotatory movements until a “give away” feel is perceived. This is caused by the entry of the punch into the semisolid subcutaneous tissue plane. The resultant wound can either be sutured or left as it is to heal by secondary intention. Areas with good vasculature, viz. face, genitals, mucosa, heal rapidly with minimal scarring.

While doing biopsy on areas where the skin is thin and overlying the bone, viz. forehead, shin, dorsum of nose or scalp, it is important not to hit the periosteum as it causes severe pain and discomfort.

Advantages of punch biopsy include ease of performance and obtaining uniformly shaped tissue. Disadvantages are that the material obtained may be inadequate and often biopsy may not include deeper tissue.

### Shave biopsy

Here, the portion of the lesion that is above the level of the surrounding skin is shaved off using a blade. Superficial lesions such as seborrheic keratosis can be biopsied in this manner. However, it is better avoided as it does not include deeper tissues.

### Saucerization biopsy

**Figure 2:**
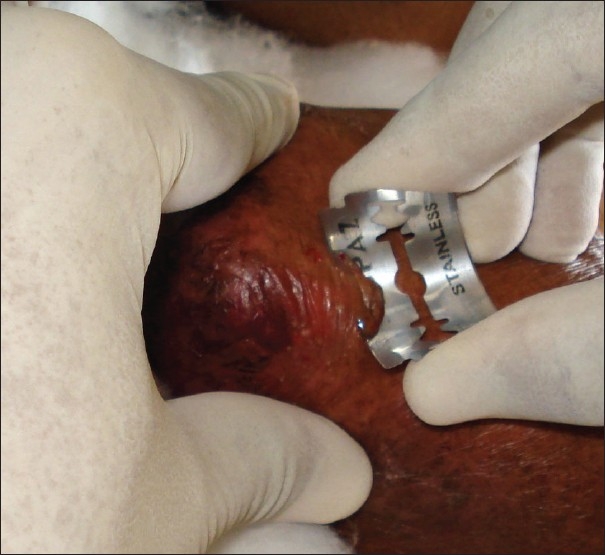
Technique of holding the blade between the index finger and the thumb to perform saucerization biopsy

Saucerization biopsy is ideal for vesiculobullous disorders and also for epidermal neoplasms like seborrheic keratosis. In this technique, the plane of cleavage passes through the reticular dermis and occasionally through the subcutaneous tissue.[6] This is performed using a shaving blade. After anaesthetizing the area, the shaving blade is held between the thumb and the index finger such that the blade is bent to form an arc [Figure 2]. The blade is introduced through the skin such that it goes through the dermis. The lesion is shaved completely in this manner and the wound is dressed to heal by secondary intention.

### Wedge biopsy

This is usually performed for large lesions where not only length and breadth but also depth of specimen is important. Hence, this technique is commonly chosen for subcutaneous mycosis, margin of squamous cell carcinoma, tuberculosis verrucosa cutis, etc.

After anaesthetizing the skin, a stab incision in a V or a triangular shape is performed with scalpel blade 11 or 21–25 so as to extract a cone of tissue. Most often the lesion is left to heal by secondary intention.

### Incisional biopsy

This involves taking a part of the tissue for confirming the diagnosis and is commonly employed when inflammatory dermatosis is suspected. The incision may extend into the surrounding normal skin. In porokeratosis, incision extends from the centre of the annular plaque through the raised margin into the surrounding normal skin so as to visualize all the three zones.

### Excisional biopsy

The entire lesion is completely and deeply removed till the subcutis plane. The wound can be closed primarily by sutures. This method is preferred when a neoplasm is suspected.

## SKIN BIOPSY IN SPECIAL CIRCUMSTANCES

- In suspected cases of urticaria pigmentosa, if local anaesthetic is infiltrated into the biopsy area, there is a risk of degranulation of mast cells which may cause difficulty in recognizing mast cells on histopathology. Hence, is such cases, field block is preferred over infiltrative anaesthesia.
- For scalp biopsy, it is important to consider the trichoglyphics. The punch biopsy should be performed in a direction parallel to the direction of emergence of hairs from the scalp. The ring of an artery forceps or needle holder can be pressed around the lesion so as to compress the vessels. This helps in reducing the bleeding during biopsy.
- When the biopsy is being performed for immunofluorescence, as in case of vesiculobullous disorders, the perilesional normal skin should be taken rather than the involved skin. The preservative for immunofluorescence is Michel’s medium and not 10% formalin. If Michael’s solution is not available, phosphate buffer saline can be used to hold the tissue and transported fresh to laboratory.
- When skin biopsy is being performed for the lupus erythematosus (LE) test, three biopsies have to be taken and the sites are lesional skin (for diagnosis of cutaneus LE), non-lesional sun-exposed skin (viz. extensor forearm for diagnosis of systemic LE) and non-lesional sun-protected skin (viz. buttocks for prognostic value to detect possibility of severe renal disease).
- In case of vascular lesions like port wine stain, haemolymphangioma, adrenaline used with local anaesthetic causes vasoconstriction and may cause difficulty in histological interpretation. Haemostasis can be achieved by suturing or cauterization.
- As with vascular lesions, biopsy from shaft or glans penis is also associated with the risk of bleeding. Current evidence does not permit use of adrenaline in these sites. Hence, haemostasis has to be achieved by suturing the wound.

## COMPLICATIONS THAT MAY BE ENCOUNTERED DURING BIOPSY

Although skin biopsy is a simple out-patient procedure, complications can occur and it is prudent to avoid them, and when they occur, to recognize them and manage them effectively [Table 1].

**Table 1:** Complications of skin biopsy, their prevention and management

Complications	Measures to prevent complications	Measures to manage complications
Hypersensitivity to local anaesthetics	Perform intradermal test or patch test before administration of local anaesthesia	Manage anaphylaxis with injection hydrocortisone, pheniramine and adrenaline if required; use alternative techniques to achieve anaesthesia
Pain of local anaesthesia	Alkalinize local anaesthetic agent; use 26 or 30 G needle; slow injection of LA; avoid overinfusion of LA; if possible, approach palmoplantar lesions from dorsal skin; cool the skin; divert the attention of the patient while injecting	
Bleeding	Avoid areas overlying vessels; use adrenaline wherever possible; use of mechanical methods, viz. chalazion clamp, to reduce perfusion	Apply pressure; use Swab soaked with hydrogen peroxide/aluminium chloride 20–40% or Monsel’s solution; suture; electrocautery
Scarring	Avoid patients with tendency for keloids/hypertrophic scars; perform subcutis deep biopsy; prevent infection with topical/systemic antibiotics	Manage keloids/hypertrophic scars as per standard guidelines; surgical excision of scar and primary suturing
Infection	Proper preparation of the area before biopsy; biopsy should be subcutis deep; use of topical/systemic antibiotics	Administer topical and/or systemic antibiotics

### Hypersensitivity to local anaesthetic agents

True hypersensitivity reactions to local anaesthetic agents, in particular amide group (lignocaine, prilocaine, bupivacaine, mepivaciane, ropivacaine), are very rare.[7] Reactions to additives often masquerade as true allergy to anaesthetic agents. An intradermal test for local anaesthetics should be performed to detect possible risk of severe type I hypersensitivity reactions. If EMLA is being used, a patch test can be performed for the same in suspected cases.

### Discomfort associated with local anaesthetic administration

Discomfort associated with administration of local anaesthetics can be due to the prick by the needle, sudden stretching of tissue due to local anaesthetic or due to the local anaesthetic agent itself. Following measures reduce the pain associated with local anaesthetic administration:

- Needles of 26 or 30 G cause minimal discomfort and are preferred over bigger needles. Pre-cooling the skin with ice cubes or application of EMLA are other options to reduce the pain of the prick.
- Sudden expansion of tissue can be prevented if only adequate and not liberal volumes of local anaesthetics are administered slowly.
- Injections on the palmoplantar aspect are exquistively painful; hence, if the lesion is close to the sides of the palms/soles, the needle can be introduced through the dorsal skin. When injecting on the palmoplantar surface, it is better to push some local anaesthetic agent, wait a while for the area to be anaesthetized and then further push the needle to reduce the discomfort.
- Local anaesthetic agents are usually acidic in pH (pH 5.0–7.0) to improve the shelf life and solubility of the agent. Hence, when injected, they give rise to a stinging sensation or pain. Solutions with adrenaline are even more acidic (pH 3.3–5.5).[8,9] This can be alleviated by combining the local anaesthetic with 8.4% sodium bicarbonate (10:1) to render the pH neutral. Reconstituted buffered lignociane with or without adrenaline can be kept at room temperature for 1 week.[10,11]

### Bleeding

Risk of bleeding is more on the scalp, face, genitals and in elderly patients with atrophic skin.

- Use of lignocaine with adrenaline helps in reducing bleeding except in few circumstances discussed in the section on “Local anaesthesia for skin biopsy”.
- Bleeding can be prevented by checking for any underlying vessels in the biopsy area and avoiding such an area.
- On the scalp, the ring of a large artery forceps can be applied with pressure around the biopsy site so that blood loss is minimized.
- Bleeding usually is due to rupture of small venules and application of pressure for about 2–3 mins stops oozing most often. Swab soaked with hydrogen peroxide or aluminium chloride 20–40% or Monsel’s solution can be used to stop bleeding. Iron present in Monsel’s solution may discolour the skin. Electrocautery can also be used to control bleeding. If the bleeding is persistent then the wound may be sutured.

### Scarring

**Figure 3:**
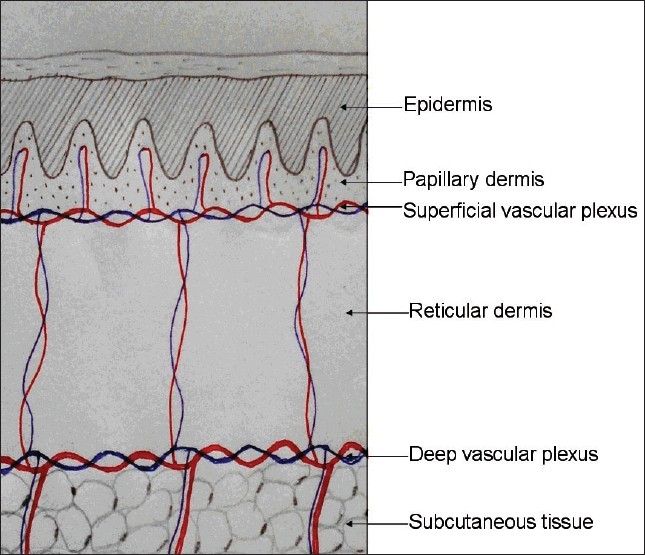
Biopsy wound heals well if it extends till the junction of the reticular dermis and subcutis where the deep vascular plexus is situated

It is prudent to rule out any history of presence of hypertrophic scars or keloidal tendency before any surgical procedure. There are two vascular plexuses in the skin; one at the junction of papillary dermis and reticular dermis and another at the junction of reticular dermis and subcutaneous tissue. These plexuses are interconnected by few vertically traversing vessels [Figure 3]. When a biopsy ends at the mid-reticular level, the residual dermis is at risk of necrosis due to reduced vascularity. This increases the chances of secondary infection and scarring. Hence, while performing biopsy, it is important to go subcutaneous tissue deep. Not only does this improve wound healing but it also promotes the semisolid subcutis to glide easily and fill the rent readily and close the wound. Primary suturing with fine sutures also reduce scarring.

### Infection

The chances of secondary infections are minimal if proper aseptic precautions are followed. In addition to the topical antibiotics, systemic agents may also be administered for persons with uncontrolled diabetes mellitus, atopics with extensive eczematization and debilitated individuals.

## PRACTICAL CONSIDERATIONS TO IMPROVE SKIN BIOPSY OUTCOME

### Optimal strength and volume of formalin solution

For fixation, the concentration of formalin should be 10%. The commercially available formalin is 40% and has to be diluted with water in a ratio of 1:4. Volume of 10% formalin required for optimal fixation is 10 times the volume of the biopsy specimen.

### Minimal handling of tissue

**Figure 4:**
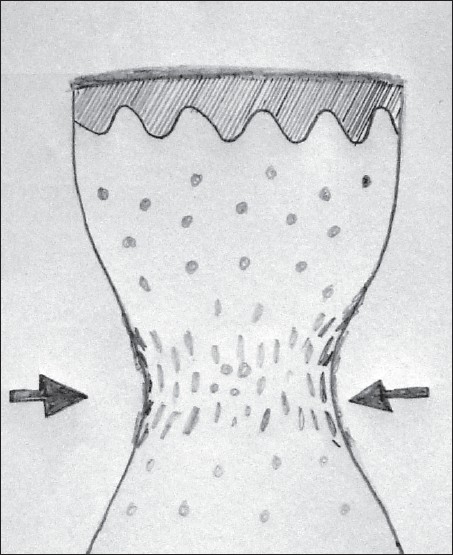
Schematic representation of the histopathologic picture of crush artifact caused by holding the biopsy tissue with forceps

**Figure 5:**
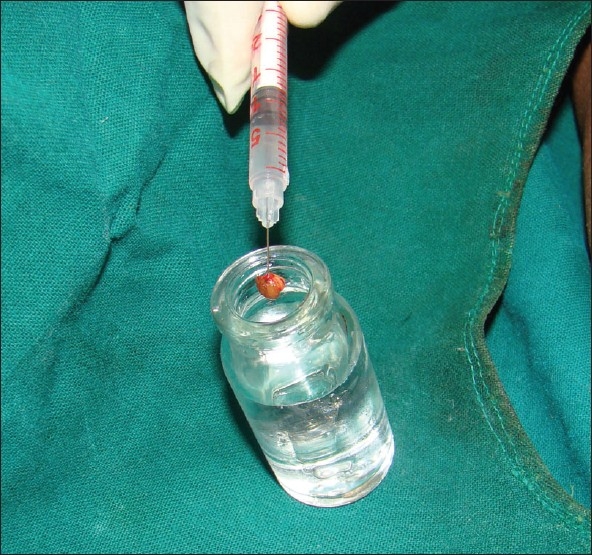
Minimal handling of tissue during transferring the biopsy specimen into formalin bulb

Transfer of tissue from the biopsy wound to the formalin container should be carried out skilfully, with minimal handling of tissue. When the tissue is crushed between the blades of forceps, the tissue is compressed laterally giving rise to compression artifact after processing [Figure 4]. Skin hook, needle of syringe used to administer local anaesthetic [Figure 5] or Jeweler’s forceps or Adson’s forceps without tooth can be used to transfer tissue atraumatically.

### Washing the specimen off excess blood

Blood collected on and around the biopsy specimen should be washed with normal saline or the residual local anaesthetic agent present in the syringe. Excess of blood around the tissue gives a false impression of vasculitis with extravasation of red blood cells on the scanner view and may mislead an amateur dermatopathologist.

### Infiltration of optimal volume of local anaesthetic agent

Overzealous infiltration of biopsy area with excess of local anaesthetic agent mimicks moderate to severe dermal oedema as in urticaria/angioedema and scleredema. This can be overcome by either reducing the amount of local anaesthetic infiltrated or by choosing field or ring-block techniques rather than infiltration anaesthesia.

## CONCLUSIONS

Skin biopsy is a simple procedure that has a great diagnostic value. Knowledge of practical aspects of this technique along with awareness of complications arising from skin biopsy and its management takes a dermatologist a long way in his dermatology practice in terms of arriving at a diagnosis or providing new and more information regarding the disease.
